# In Situ Synthesis of a Silver-Containing Superabsorbent Polymer via a Greener Method Based on Carboxymethyl Celluloses

**DOI:** 10.3390/molecules23102483

**Published:** 2018-09-27

**Authors:** Jie Shen, Chang Cui, Jian Li, Lijuan Wang

**Affiliations:** 1 Key Laboratory of Bio-Based Materials Science and Technology of Ministry of Education, Northeast Forestry University, Harbin 150040, China; sj@nefu.edu.cn (J.S.); 15114532032@163.com (C.C.); nefulijian@163.com (J.L.); 2 Research Center of Wood Bionic Intelligent Science, Northeast Forestry University, Harbin 150040, China

**Keywords:** superabsorbent polymer, silver particles, carboxymethyl cellulose, antibacterial property

## Abstract

An antibacterial superabsorbent polymer (SAP) was synthesized by grafting acrylic acid (AA) onto carboxymethyl cellulose (CMC) and mixing with silver particles, with *N*,*N*′-methylenebisacrylamide used as a crosslinker and potassium persulfate as an initiator. Silver nanoparticles were produced through the reaction between glucose and silver nitrate. The effects of the amount of silver nitrate added in the polymer on the swelling ratio were investigated and the maximum swelling ratio of the SAP loaded with silver particles in distilled water and in a 0.9 wt % NaCl solution reached 840 g/g and 71 g/g, respectively, when the silver nitrate added was 50 mg. The SAP was characterized by Fourier transform infrared spectroscopy, X-ray photoelectron spectroscopy, ultraviolet-visible spectroscopy, scanning electron microscopy, energy dispersive spectrometry, transmission electron microscopy, and thermogravimetric analysis. Through these analysis methods, it could be seen that the acrylic acid was successfully grafted onto CMC, forming a three-dimensional network structure, with the successful production of silver nanoparticles with sizes ranging from 5 nm to 50 nm. Moreover, the antibacterial properties of the SAP loaded with silver nanoparticles against *Staphylococcus aureus* and *Escherichia coli* were investigated and the results show that they became more effective with increasing silver nitrate concentration. The obtained SAP can be useful in developing new antibacterial medical and public health supplies.

## 1. Introduction

Superabsorbent polymers (SAPs) are a kind of three-dimensional cross-linked hydrophilic polymer that can absorb and retain large amounts of water or aqueous solution due to their networks and hydrophilic groups, such as hydroxyl and carboxyl groups [1,2]. Owing to their excellent water absorbency, the applications of SAPs are continuing to grow in a variety of fields, such as in waste disposal, agriculture, the food industry, hygiene products, and consumer products [3,4,5,6]. Although SAPs have been widely used and commercialized, it is still essential to broaden the use of SAPs and to improve their performance, including their liquid-absorbing capacity and antibacterial properties. Furthermore, there are still a number of challenges that must be addressed in these fields, meaning that additional research needs to be carried out.

An important issue is that most commercialized SAPs are synthetic polymers based on vinyl monomers, such as acrylic acid and acrylamide, which are poorly biodegradable and harmful to the environment [7]. To solve this problem, significant research has been completed to produce eco-friendly SAPs based on natural materials to reduce the damage to the environment. As expected, natural materials are of particular interest because they can be biodegradable and are both inexpensive and widely available. Hence, polysaccharides and proteins, such as guar gum, tara gum, tragacanth gum, starch, cellulose, and soy protein, have become a promising focus in the design and preparation of natural-based SAPs because these carbohydrate polymers are biodegradable, biocompatible, nontoxic, and economical [8,9,10,11,12,13]. Researches have shown that the copolymerization between vinyl monomers and natural materials can be conducted successfully [14,15].

Carboxymethyl cellulose (CMC) is a biodegradable and biocompatible polymer that can be widely used in preparing eco-friendly SAPs. It is a representative cellulose derivative of some hydroxyl carboxylated groups on the cellulose backbone and is usually made by the alkali-catalyzed reaction of natural cellulose with chlorinated acetic acid [16]. It has been widely used as a thickener and stabilizer in the food industry [17,18]. Therefore, it is a good choice for the preparation of natural-based SAPs.

More than 80% of SAPs in the world are currently used in public health products and cosmetics in which antibacterial properties are required [19,20]. Therefore, in order to improve and expand the applications of SAPs for medical and public health usage, it is very important to enhance their antibacterial properties. To improve the antibacterial activity, silver nanoparticles have been added as an antibacterial agent [21]. Among the various antimicrobial agents, metal particles show a broad spectrum of antibacterial activity such as silver, titanium dioxide, and zinc oxide nanoparticles [22,23]. Silver nanoparticles have unique electrical, optical, catalytic properties and antibacterial activity, resulting in numerous applications in industrial and medical fields, such as water treatment, bio-sensing, and the treatment of disease [24,25,26]. Silver is the most effective antibacterial metal particle and it shows effective antimicrobial efficacy against bacteria, fungi, and viruses [27,28]. On the one hand, silver can react with the sulfur-containing and amine-containing functional groups in nucleic acids and proteins, such as -SH and -NH_2_, leading to protein coagulation and enzyme inhibition. On the other hand, silver reacts with the DNA of microorganisms, destroying the normal activities of some functional systems in the cell and thereby preventing normal metabolism [29,30]. Currently, silver has widely been used as an effective bacteriostat in wound dressings, medical products, and water purification systems [31,32]. In recent years, studies on silver nanoparticles-loaded SAP have developed rapidly. In many studies, an in situ synthesis method was used with trisodium citrate (C_6_H_5_O_7_Na_3_) [33] and sodium borohydride (NaBH_4_) [34] as reducing agent, which is costly and may introduce pollution from the other synthetic chemicals. Juby et al. synthesized a silver nanoparticles-loaded SAP with γ-rays which are harmful to the human body [35]. Therefore, simpler and greener methods for synthesizing silver nanoparticles-loaded SAP are required. Glucose is an important monosaccharide widely distributed in nature and used as a reducing agent because of the aldehyde group [36,37]. It is an excellent choice for the preparation of silver nanoparticles because of its wide source, low cost, with no pollution to the environment.

In the current work, a silver nanoparticles-loaded SAP based on a CMC graft copolymerized with PAA was synthesized by in situ synthesis. Silver nanoparticles were produced with glucose reducing silver nitrate. We investigated the effect of the amount of silver nitrate (AgNO_3_) added in the polymer on its swelling ratio in distilled water and 0.9 wt % NaCl solution. The polymers were characterized using Fourier transform infrared spectroscopy (FTIR), X-ray photoelectron spectroscopy (XPS), X-ray diffraction (XRD); ultraviolet-visible spectrophotometry (UV-Vis), scanning electron microscopy (SEM), energy dispersive spectrometry (EDS), and thermogravimetric analysis (TGA). Furthermore, we tested the antibacterial activity of the SAP loaded with silver nanoparticles against Staphylococcus aureus and Escherichia coli. The results above are valuable and useful for the understanding and application of this type of SAP in medical applications.

## 2. Materials and Methods

### 2.1. Materials

Carboxymethyl cellulose (CMC) was obtained from Dymatic Fine Chemical Co., Ltd. (Guangzhou, China). Acrylic acid (AA) was purchased from Tianli Chemical Reagent Co., Ltd. (Tianjin, China), purified by activated carbon before use. Sodium hydroxide (NaOH) was purchased from Guangfu Technology Development Co., Ltd. (Tianjin, China). Potassium persulfate (KPS) was provided by Sitong Chemical Plant (Tianjin, China). *N*,*N*-methylene bisacrylamide (MBA) was provided by Regent Chemicals Co., Ltd. (Tianjin, China). Glucose was provided by Yongda Chemical Reagent Co., Ltd. (Tianjin, China) and silver nitrate (AgNO_3_) was purchased from Fine Chemical Materials Research Institute (Shanghai, China). *Staphylococcus aureus* (*S. aureus*) and *Escherichia coli* (*E. coli*) were purchased from Haibo Co., Ltd. (Shandong, China).

### 2.2. Synthesis of CMC-g-PAA Superabsorbent Hydrogel Loaded with Silver Particles

Activated carbon was added to crude AA to remove the polymerization inhibitors and impurities. After standing for 1 h and filtering, the purified AA was obtained. A mixed solution containing purified AA, NaOH solution (2 mol/L), MBA (6.49 × 10^−2^ mol/L), and glucose (1.11 × 10^−1^ mol/L) was prepared by agitation in an ice bath. Then 1 g CMC and 20 mL distilled water were added with stirring into a 3-neck round bottom flask under nitrogen atmosphere and the flask was placed in the water bath at 60 °C. AgNO_3_ (5.89 × 10^−2^ mol/L) was added into the flask after 10 min stirring and the solution of initiator (KPS, 7.40 × 10^−2^ mol/L) was added after another 10 min. After stirring 15 min, the mixed solution was added to the flask under the protection of nitrogen as before. The molar ratio of glucose to silver nitrate was 2:1 to ensure that silver ions react fully to form silver particles. The hydrogel was obtained after another 75 min stirring. The polymer was cut into small pieces and dried at 60 °C after immersion cleaning with ethanol. The dried product was crushed into a certain size of granularity for characterization and test.

### 2.3. Characterization

#### 2.3.1. Swelling Behavior

A certain amount of 80–120 mesh SAP powder (about 0.1 g) was placed into a 200-mesh nylon sieve pouch and then immersed into a certain weight of distilled water (300 mL) or 0.9 wt % NaCl solution (200 mL) to reach the swelling equilibrium at room temperature. The pouch with SAP powder was taken out from water after reaching the swelling equilibrium and then stood for 30 min to filter out the excess water. The swelling ratio of the sample was calculated by the equation as follows:

Q = (m_1_ − m_2_ − m_3_)/m_3_ (1)

where m_1_ is the weight of the swollen SAP (g), m_2_ is the weight of the nylon sieve pouch (g) and m_3_ is the weight of the dry SAP (g). Q is the swelling ratio (g/g) of the SAP [38].

#### 2.3.2. FTIR Spectroscopy

FTIR analysis was recorded on a Nicolette 6700 spectrometer (Thermo Fisher Scientific Co., Ltd., Waltham, MA, USA) with attenuated total reflection (ATR) mode in the range of 4000–600 cm^−1^ at a resolution of 4 cm^−1^.

#### 2.3.3. X-ray Diffraction (XRD)

XRD patterns of the hydrogels were analyzed by using a D/max-2200 diffractometer (Rigaku, Japan) at a voltage of 40 kV and a current of 30 mA through Cu-Kα radiation. The scanning scope of 2θ was ranged from 5° to 90° at a scanning rate of 5°/min. The sample was ground to a powder of >200-mesh for testing.

#### 2.3.4. X-ray Photoelectron Spectroscopy (XPS)

X-ray photoelectron spectroscopy (XPS) was carried out on a Kratos Axis Ultra DLD X-ray photoelectron spectrometer with an Al Kα X-ray source (1486.6 eV photons).

#### 2.3.5. UV-Vis Spectrophotometer

The UV-Vis absorption spectra of the SAPs were performed on an ultraviolet-visible (UV-Vis) spectrophotometer (UV-2600, Shimadzu, Kyoto, Japan) with a scan range of 200–600 nm. Silver particles have unique and tunable optical properties due to surface plasmon resonance (SPR) and this is related to the shape, size, and size distribution of the particles [39]. In this study, SAPs were tested after swelling in water. The distilled water was used as blank solution.

#### 2.3.6. Scanning Electronic Microscopy (SEM)

Surface morphology of the samples was examined on a Quanta 200 scanning electron microscope (Philips-FEI Co., Amsterdam, The Netherlands). Samples coated with a thin gold layer were analyzed at an accelerating voltage of 10 kV.

#### 2.3.7. Energy Dispersive Spectrometer (EDS)

The element analysis of the particles in the superabsorbent polymer was carried out by SEM equipped with an energy dispersive spectrometer which can provide a rapid qualitative and quantitative analysis of the elemental composition.

#### 2.3.8. Transmission Electron Microscopy (TEM)

The shape and size of silver nanoparticles were characterized through transmission electron microscopy (TEM) by using the JEM-2100 (JEOL LTD, Tokyo, Japan) at 200 kV. The freeze-dried hydrogel was dispersed in ethanol. Two to three drops of the dispersion were dispensed onto the grid and the excess fluid was removed after 5 min. The air-dried grid was then observed by using TEM.

#### 2.3.9. Thermogravimetric Analysis (TGA)

Thermal stability of the prepared hydrogels was implemented using a TA Instruments TGA Q500 thermal gravimetric analyzer (TA Instruments, New Castle, DE, USA). The samples were heated from 25 to 600 °C at a rate of 20 °C/min under nitrogen atmosphere. A derivative form of TGA (DTG) was obtained.

#### 2.3.10. Antibacterial Activity

Antibacterial activity of the superabsorbent hydrogels was determined against both Gram-positive bacteria, *S. aureus* and Gram-negative bacteria, *E. coli* according to the agar diffusion test [40]. The superabsorbent polymers were pressed into tablets of 1 cm after grinding into powder and placing the tablets on the agar plate to expose to bacteria in solid media. The inhibition zone around each tablet was measured and recorded as the antibacterial effect of SAPs after the agar plates with SAPs were incubated at 37 °C for 12 h.

## 3. Results and Discussion

### 3.1. Reaction Mechanism for Formation of the SAP Loaded with Silver Particles

Graft copolymerization of acrylic acid onto CMC was carried out in the presence of KPS as an initiator and MBA as a crosslinking agent. In order to initiate the grafting process, the initiator was first decomposed by heating to 60 °C to produce free radicals which further initiated CMC to form alkoxy groups. The monomer molecules that are closely adjacent to free radicals accept CMC free radicals, leading to chain initiation reactions. As a result, the monomer molecules become free radical donors of adjacent molecules to support graft chain growth and a chain structure formed with a considerable molecule weight [41]. Because of the presence of the crosslinking agent, MBA, the chain structures polymerized into a network structure.

**Scheme 1:**
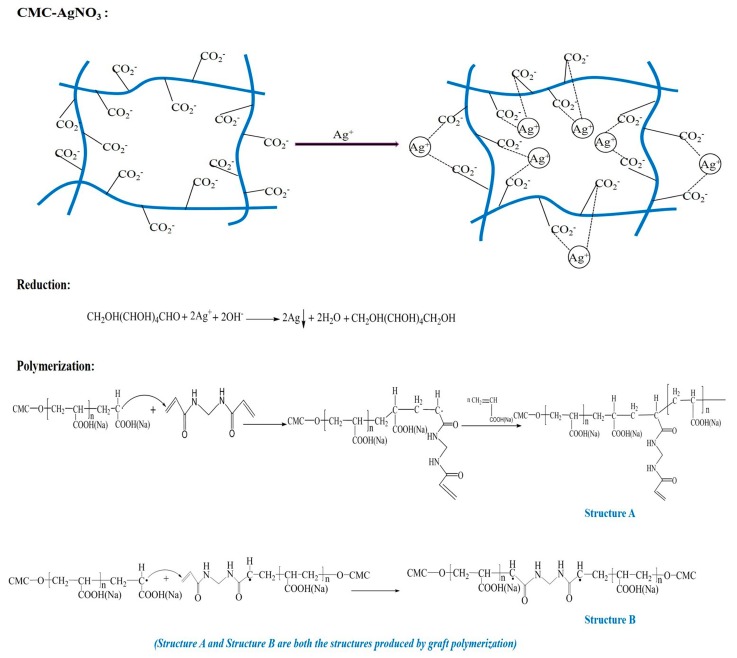
The mechanism for preparation of silver nanoparticles-loaded superabsorbent polymers (SAPs).

Since silver is a transition metal with empty orbitals, it can form carboxylate metal ion complexes [42]. The silver nanoparticles are immobilized throughout the networks because of the strong localization of the silver ions within the network, which is caused by the complexation of silvers ions by the carboxyl groups [21] and polymers with carboxylic acid groups have the ability to stabilize compounds with metal ions [43]. CMC contains carboxyl groups that can stabilize the silver ions. Then, the added glucose can reduce silver ions to silver nanoparticles. Scheme 1 shows the procedure of the preparation of the SAP loaded with silver nanoparticles.

### 3.2. Swelling Behavior

**Figure 1:**
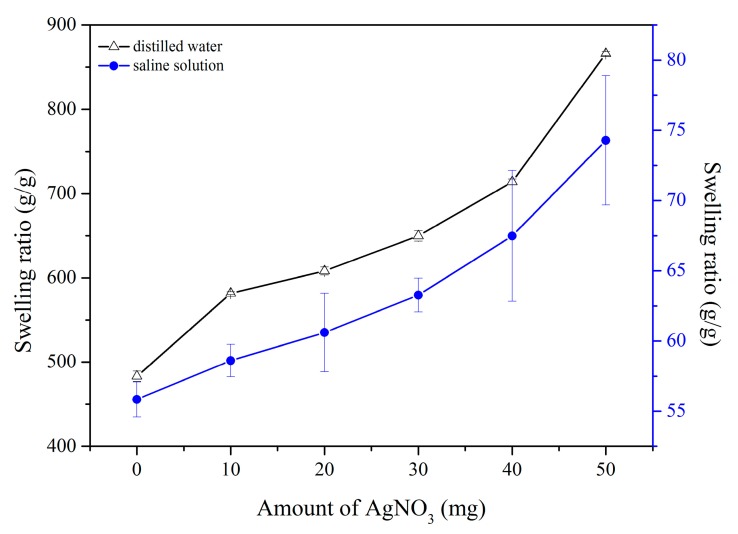
Swelling behavior in distilled water and 0.9 wt % NaCl solution of the superabsorbent polymer (SAP).

Figure 1 shows the swelling behavior of the SAP prepared in distilled water and 0.9 wt % NaCl solution. It can be seen that with an increasing amount of AgNO_3_, the swelling ratio of the SAPs increases. The water absorption capacity of the SAP is mainly due to its three-dimensional network structure and hydrophilic groups. When there are no silver nanoparticles in the SAP, its swelling ratio can reach 480 g/g in distilled water and 55 g/g in 0.9 wt % NaCl solution. However, when the silver particles are embedded in the SAP, the swelling ratio increased significantly. The swelling ratio of CMC-g-PAA/Ag with 50 mg AgNO_3_ added in distilled water and 0.9 wt % NaCl solution reached 860 g/g and 74 g/g, respectively, which is much higher than some other SAPs [44,45]. The reason for this may be that the formation of silver nanoparticles can increase the pores and free spaces within the network structure of the SAP, and as a consequence, the SAP can adsorb more water than before.

### 3.3. FTIR Analysis

**Figure 2:**
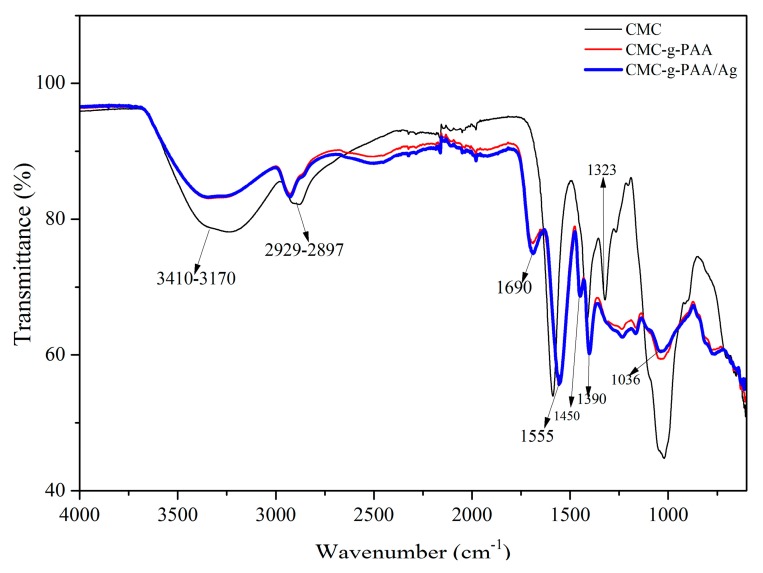
Fourier transform infrared spectroscopy (FTIR) spectra of carboxymethyl cellulose (CMC), CMC-g-PAA and CMC-g-PAA/Ag.

The FTIR spectra of CMC, CMC-g-PAA and SAP loaded with silver nanoparticles are shown in Figure 2. In the spectrum of CMC, the broad band at 3420 cm^−1^ is assigned to the stretching vibration of O–H, which is associated with free, inter and intra-molecular bound hydroxyl groups [46]. The bands at about 2929 cm^−1^ and 2897 cm^−1^ were attributed to the C–H stretching vibration of hydrocarbon [47]. Characteristic bands at 1590 cm^−1^ and 1420 cm^−1^ are due to the symmetric and asymmetric stretching of COO^−^ groups in CMC, respectively [48]. The peak at 1323 cm^−1^ is attributed to the stretching of O–H bending, respectively [49] and the absorption band at 1024 cm^−1^ indicated the C–O–C stretching of 1,4-b-d-glucosidic linkage [50]. For the spectra of CMC-g-PAA and CMC-g-PAA/Ag, the peaks at ~3420 cm^−1^ and ~1323 cm^−1^ (O–H group) and the peak at ~1024 cm^−1^ (C–O–C group) become smaller, which is due to the graft copolymerization between CMC and PAA. The new band at 1690 cm^−1^ is attributed to the C=O stretching vibration of the carboxyl group in PAA, which means the PAA was grafted onto the CMC successfully [51]. The peak at 1555 cm^−1^ is due to the COO^−^ group. Since the carboxyl groups in the CMC coordinate with Ag^+^ and the acrylic acid used in the synthesis of SAP also has COO^−^ groups, the peak is strong and red-shifted. The peaks appearing at 1450 cm^−1^ and 1380 cm^−1^ are caused by the vibrational absorption of –CH_3_ of the SAP. The weak bands at 1236 cm^−1^ and 1170 cm^−1^ are due to the C–O–C stretching vibration. The characteristic features of the spectrum of SAP loaded with silver particles are almost similar to the spectrum of the CMC-g-PAA because silver nanoparticles do not have characteristic peaks in FTIR.

### 3.4. XRD Analysis

**Figure 3:**
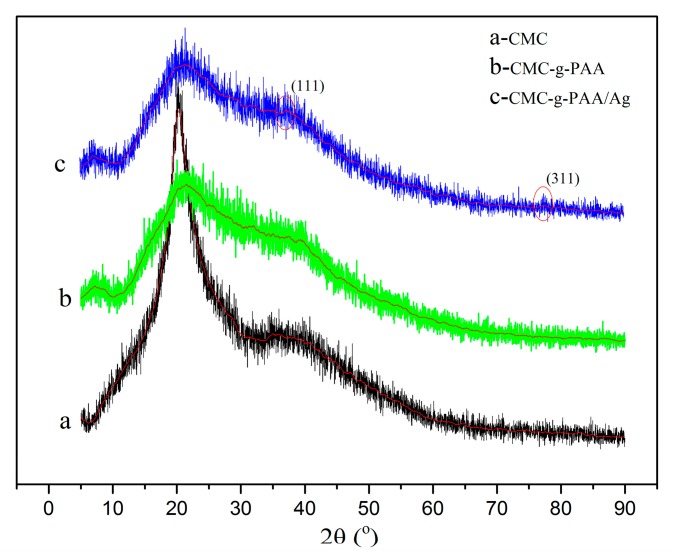
X-ray diffraction (XRD) patterns of CMC, CMC-g-PAA and CMC-g-PAA/Ag.

Figure 3 displays the XRD patterns of CMC, CMC-g-PAA and CMC-g-PAA/Ag. A sharp peak at ~20° indicates that amorphous and crystalline regions existed in CMC, which became weakened in the patterns of CMC-g-PAA and CMC-g-PAA/Ag, indicating that the graft copolymerization between PAA and CMC destroyed the ordered arrangement of CMC. After embedding silver nanoparticles, the XRD pattern of CMC-g-PAA/Ag is similar to that of CMC-g-PAA, while peaks at ~37° and ~77° occurred due to the (111) and (311) planes of the silver nanoparticles [45].

### 3.5. XPS Analysis

**Figure 4:**
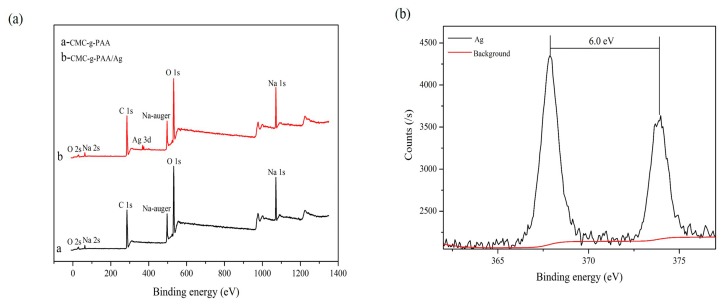
X-ray photoelectron spectroscopy (XPS) spectra of (**a**) CMC-g-PAA and CMC-g-PAA/Ag, (**b**) XPS peak of Ag (3d) in CMC-g-PAA/Ag.

XPS was carried out to determine the chemical state of silver in the SAP. Figure 4a,b show the results of the XPS wide sweep of CMC-g-PAA and CMC-g-PAA/Ag. Upon comparing the two graphs, there is a clear 3d peak of silver in Figure 4a, which shows the presence of metallic silver in CMC-g-PAA/Ag. In addition, Figure 4b shows high-resolution XPS spectra of Ag (3d) core levels in CMC-g-PAA/Ag. The binding energies for Ag (3d_5/2_) and Ag (3d_3/2_) peaks are 368.0 eV and 374.0 eV, respectively, whereas the binding energies shown in Figure 4b are 367.9 eV and 373.9 eV, respectively. The 3d double cleavage of Ag is 6.0 eV, indicating the formation of metallic silver in the SAP [52,53].

### 3.6. UV-Vis Spectrophotometer

**Figure 5:**
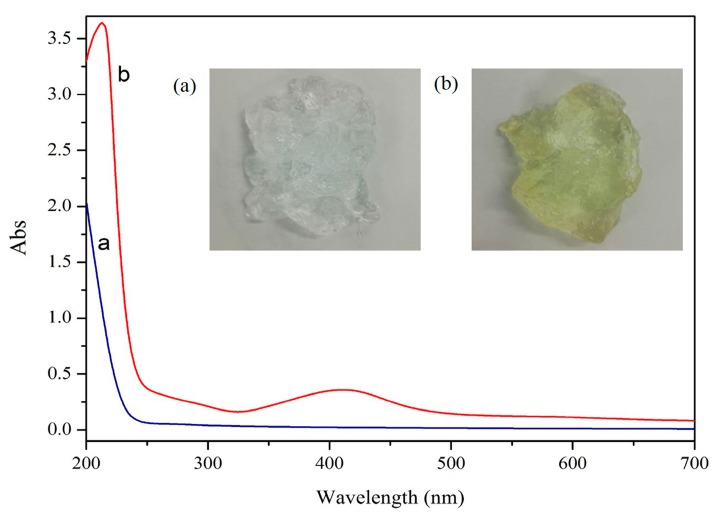
UV-Vis spectra of (**a**) CMC-g-PAA and (**b**) CMC-g-PAA/Ag.

The UV-Vis spectra of CMC-g-PAA and CMC-g-PAA/Ag are shown in Figure 5. Compared to the spectrum of CMC-g-PAA, the spectrum of CMC-g-PAA/Ag has a distinct peak at about 420 nm. The peak at 420 nm corresponds to the wide SPR of the silver particles, confirming the formation of silver particles with a broad size distribution in the SAP [54]. The band shows the size distribution of the silver particles [55].

### 3.7. Morphologies of the Superabsorbent Polymers

**Figure 6:**
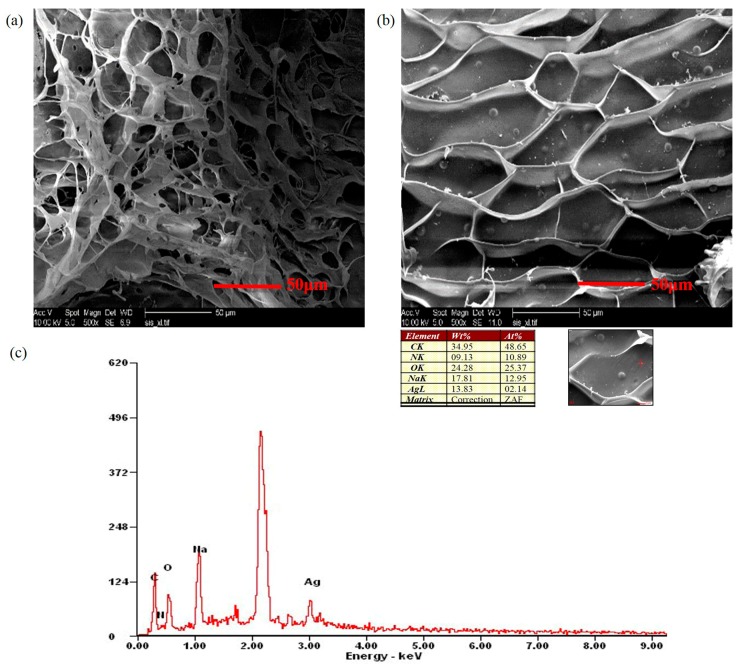
Scanning electron microscopy (SEM) of (**a**) CMC-g-PAA, (**b**) CMC-g-PAA/Ag, and (**c**) energy dispersive spectrometry (EDS) of CMC-g-PAA/Ag.

The water absorbency and retention rate of the SAP depend on the hydrogel porosity and pore size [56]. An important property of SAPs is their microstructure morphologies. As shown in Figure 6, the SEM micrographs of the CMC-g-PAA and CMC-g-PAA/Ag depicted a developed 3D network structure in which the size of the pores are about 45–50 μm providing spaces for water absorption. It can be seen from Figure 6a,b that the SAP has a three-dimensional network and large amounts of pores, which is due to the graft copolymerization between AA and CMC. After absorbing water, the compact network structure fully expands and the suction space becomes larger. In addition, the generation of silver particles in CMC-g-PAA/Ag results in more pores, which corresponds to the increase in the water absorbency of the SAP.

The EDS analysis for SAP loaded with silver particles is shown in Figure 6c. The EDS quantitative analysis shows that the SAP loaded with silver particles (50 mg of silver nitrate) contains about 9.13 wt % nitrogen, 13.83 wt % silver, 17.81 wt % sodium, 24.28 wt % oxygen and 34.95 wt % carbon. The existence of nitrogen and sodium is the result of crosslinking agent and NaOH, while the existence of silver is due to the reaction between glucose and silver nitrate. The presence of silver endowed the SAP with antibacterial properties shown in Figure 6.

### 3.8. Transmission Electron Microscopy (TEM)

**Figure 7:**
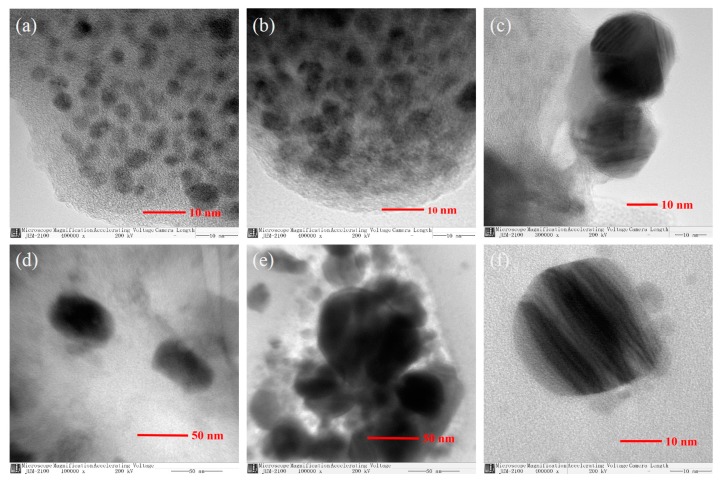
Transmission Electron Microscopy (TEM) images of the silver nanoparticles when the amount of AgNO_3_ is (**a**) 10 mg; (**b**) 20 mg; (**c**) 30 mg; (**d**) 40 mg; (**e**) 50 mg, and (**f**) a single silver nanoparticle.

As can be seen in Figure 7, the silver nanoparticles exist in irregular nanospheres with sizes ranging from 5 nm to 50 nm. The size of the particles gradually increased as the amount of AgNO_3_ increased because the increased amount of silver makes the particles conglomerate. Therefore, larger particles formed.

### 3.9. Thermal Stability

**Figure 8:**
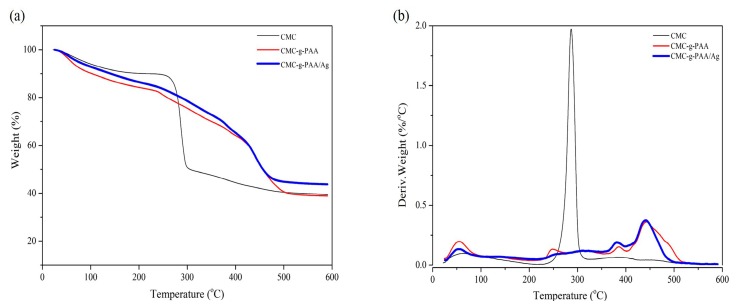
Thermogravimetric (TG) (**a**) and DTG (**b**) curves of CMC, CMC-g-PAA and CMC-g-PAA/Ag.

The TGA thermograms of CMC, CMC-g-PAA, and CMC-g-PAA/Ag, along with their DTG curves, are shown in Figure 8, which can accurately indicate the temperature changes of the hydrogel in the application. The TGA curves showed the weight loss pattern and the DTG curves showed the maximum decomposition temperature for each thermal degradation step. It can be seen from Figure 8 that CMC, CMC-g-PAA, and CMC-g-PAA/Ag all undergo multi-step thermal degradation. CMC has two stages of weight loss. The first weight reduction is mainly due to the small amount of moisture in the sample. The second stage is mainly due to the decomposition of CMC. As CMC has carboxyl groups and in this temperature range decarboxylation occurs, this part weight loss is because of the CO_2_ loss from polysaccharides. According to CMC-g-PAA, the first step occurs at 80–230 °C, which is mainly due to the moisture and residual organic solvent in the SAP. The second stage is at 250–400 °C and it is mainly because of the decomposition of linear small molecules and oligomers. The third stage is mainly due to the decomposition of the SAP. The TGA curve of CMC-g-PAA/Ag is similar to that of CMC-g-PAA. The only difference is that in the third weight loss stage, the weight loss of CMC-g-PAA/Ag is less than CMC-g-PAA in the third stage. This is because CMC-g-PAA/Ag contains silver particles that cannot be broken down in a nitrogen atmosphere.

### 3.10. Antibacterial Activity

**Figure 9:**
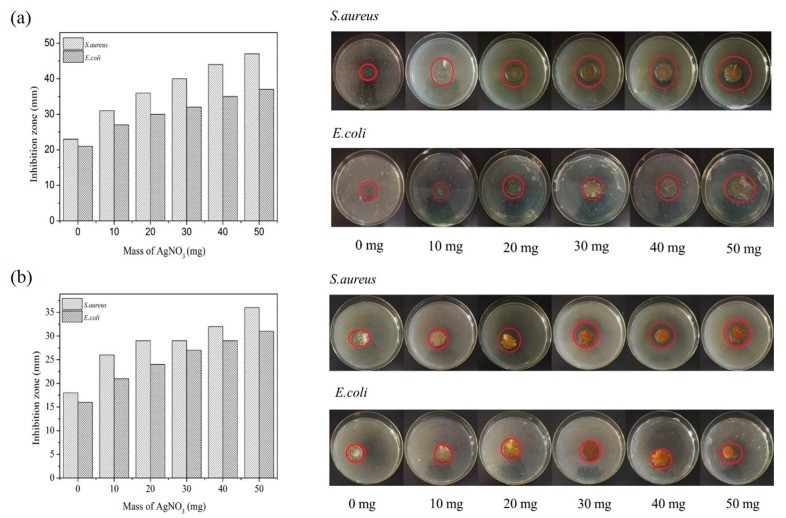
The antibacterial activity of the samples (**a**) and the dried samples after swelling in distilled water (**b**).

The antibacterial activity of the SAP loaded with silver nanoparticles was measured against Gram +ve bacteria (*S. aureus*) and Gram −ve bacteria (*E. coli*) using an agar diffusion test. It can be seen from Figure 9a that the SAPs containing silver nanoparticles have great antibacterial properties against both *S. aureus* and *E. coli*, as evidenced by the larger inhibition zone while the effect of inhibiting *S. aureus* is better than that of *E. coli*. This can be attributed to the fact that silver nanoparticles can interact with phosphorus-containing compounds and sulfur-containing proteins from the cell membrane, attacking the respiratory chain and cell division, leading to cell death. It can also be seen that with increasing AgNO_3_, the antibacterial effect improves because the silver content increased, thus enhancing the antibacterial effect. Some other studies also proved that silver nanoparticles used in biomass composites exhibit good antibacterial activity [57,58].

The dried SAPs still have an antibacterial effect after swelling in water, as can be seen from Figure 9b. Some silver nanoparticles did not escape from the network due to the packaging effect and electrostatic effect. Therefore, the SAP-containing silver nanoparticles still show certain antibacterial effects against *S. aureus* and *E. coli*.

## 4. Conclusions

In this work, an antibacterial SAP based on CMC grafted with PAA and loaded with silver nanoparticles was successfully prepared with improved water absorbency and good antibacterial activity. The structure of the silver nanoparticles-loaded SAP was confirmed by FTIR, XRD, XPS, UV-Vis, SEM, EDS, TEM, and TGA analysis. From the swelling test, it can be seen that the maximum swelling ratios in distilled water and 0.9 wt % NaCl solution were 860 g/g and 74 g/g, respectively. The results revealed that PAAs were well grafted on CMC molecules and silver nanoparticles were successfully synthesized in the antibacterial SAPs. The SEM micrographs show that the SAPs have a porous structure and three-dimensional network that can accommodate a large amount of water. In addition, it can be seen from the TGA analysis that the thermal stability of the antibacterial SAP increased after adding silver nanoparticles and it can be safely used up to 300 °C. Moreover, it can be seen clearly from the results of the antibacterial activity test that this type of SAP has a significant and effective inhibitory effect on *S. aureus* and *E. coli*. Therefore, this type of SAP can be widely used in pharmaceutical and public health products.
